# Epidemiological characteristics and factors affecting healing in unintentional pediatric wounds

**DOI:** 10.3389/fpubh.2024.1352176

**Published:** 2024-05-23

**Authors:** Hua Gao, Yang Li, Shaobin Jin, Wenli Zhai, Yanhua Gao, Linzhe Pu

**Affiliations:** ^1^Department of Nursing, Qilu Hospital of Shandong University, Jinan, Shandong, China; ^2^Department of Pediatric Surgery, Qilu Hospital of Shandong University, Jinan, Shandong, China

**Keywords:** children, unintentional injury, skin laceration, poor wound healing, epidemiology

## Abstract

**Objective:**

To analyze the epidemiological characteristics and wound healing conditions of common unintentional skin lacerations in children.

**Methods:**

A retrospective analysis was conducted on data from 1,107 children, aged 0–12 years, with skin lacerations who received emergency treatment at Qilu Hospital of Shandong University from January 1, 2019, to December 30, 2022. Data on age, injury site, time from injury to suturing, and wound healing conditions were statistically analyzed.

**Results:**

Among the 1,107 cases, 714 (64.5%) were male and 393 (35.5%) were female, with a male-to-female ratio of 1.8:1; median age was 5 years (IQR, 3–7). Infants and toddlers (0–3 years old) constituted the highest proportion, accounting for 36.3% (402 cases). The number of children aged over 3 years gradually decreased with increasing age. In younger children, the most common injuries were to the forehead, scalp, and lower jaw; in school-aged children, the proportion of limb and trunk injuries significantly increased. Age (OR, 1.34; 95% CI, 1.23–1.46), outdoor injuries (OR, 2.21; 95% CI, 1.18–4.16), lower limb injuries (OR, 5.35; 95% CI, 2.86–10.00), and wound length greater than 3 cm (OR, 10.65; 95% CI, 5.02–22.60) were significant risk factors for poor wound healing. The risk of poor wound healing increased by 34% for each additional year of age.

**Conclusion:**

In children, the common sites of unintentional skin lacerations show distinct age and gender distribution characteristics. Older age, outdoor injuries, longer wound lengths, and lower limb injuries are independent risk factors for poor wound healing.

## Introduction

Childhood accidental injuries have always been a global public health concern. According to statistics from the World Health Organization (WHO), more than 10 million children worldwide require hospital treatment for unintentional injuries each year, with 950,000 resulting in fatalities (1). In 2020, unintentional injuries accounted for 14.7% of all child deaths in China, with a incidence of approximate 1 per 10,000 children per year (2). Despite societal advancements, unintentional injuries remain a major threat to the health of children and adolescents (1–3).

Children’s skin wound account for 12.3% of unintentional injuries (1). Although most skin wound are non-fatal, they still bring direct or indirect economic burdens and social costs, causing permanent aesthetic and functional complications in children (4, 5). A study on children’s facial injuries found that 2.8% of the children experienced wound infection or significant scar hyperplasia (6). Additionally, to effectively treat trauma, medical institutions need to understand the epidemiological characteristics of trauma, including common types of injuries and treatment outcomes. However, current research on the epidemiological characteristics and prognosis of common skin injuries in children is limited. Therefore, we compiled clinical data on children with unintentional skin injuries treated at our hospital over the past 4 years to analyze their epidemiological characteristics, treatment outcomes, and factors affecting wound healing. This may provide a reliable basis for improving treatment efficiency, formulating prevention strategies, and informing prognosis.

## Materials and methods

### Cases

This study was conducted at Qilu Hospital of Shandong University, the largest general hospital and regional medical center in Shandong Province, Eastern China. The hospital boasts a capacity of 4,360 beds and accommodates more than 3 million outpatient visits annually, covering over 60 clinical and technical departments. This broad range of services underscores the hospital’s substantial role in providing emergency and specialized care in the region.

This study was approved by the Ethics Committee of Qilu Hospital of Shandong University, and adheres to the 1975 Declaration of Helsinki. As this was a retrospective study of de-identified patients, informed consent from guardians and children was waived by the Ethics Committee.

The subjects were children with skin lacerations who received suturing at the emergency department of Qilu Hospital of Shandong University from January 1, 2019, to December 30, 2022. Cases were identified by a comprehensive search of the institutional database. Inclusion criteria were: (1) age ≤ 12 years at the time of presentation; (2) with skin laceration requiring suturing. Exclusion criteria included: (1) life-threatening injuries such as cranial brain injuries, abdominal and thoracic organ damage, open fractures; (2) superficial injuries not involving the dermis.

### Data collection

Two authors collected data, including age, gender, location of the injury (outdoor or indoor), wound site, wound length, time from injury to treatment, and wound healing conditions, from the electronic medical records. The collected data were cross-checked for accuracy. Any inconsistencies identified during this process were discussed and the original medical records were re-checked to resolve these discrepancies.

### Grouping

To analyze the epidemiological characteristics of injuries in different age, the study divided children into four age groups: 0–3 years, 4–6 years, 7–9 years, and 10–12 years. Injury sites were categorized as scalp, forehead (from the eyebrow to the hairline), lower jaw, face and neck (from below the eyebrow level to the clavicle, excluding the lower jaw), upper limbs, trunk and perineum, and lower limbs. Wound lengths were grouped into <1 cm, 1–3 cm, and > 3 cm; and based on the time from injury to suturing into: 0–2 h, 2–4 h, 4–8 h, and > 8 h. The qualifications of the medical professionals who performed the suturing procedures were categorized into three groups based on their years of experience: less than 2 years, 2–5 years, and more than 5 years.

### Outcomes

The study’s outcomes included the epidemiological characteristics of children’s skin lacerations and instances of poor wound healing. Poor wound healing was defined as wound exudation, wound rupture, scar hyperplasia, sinus formation, and skin or flap necrosis (7).

### Statistical analyses

Statistical analyses were conducted using SPSS for Windows, version 26.0 (IBM., Armonk, NY, United States). Continuous variables were expressed as the median (interquartile range, IQR) and analyzed using the Mann–Whitney U test; categorical variables were expressed as numbers (percentage) and analyzed using the Chi-square test or Fisher’s exact test, as appropriate. Risk factors for poor wound healing were analyzed using univariable analysis, which incorporated gender, age, location of injury, injury site, wound length, and time from injury to suturing. Adjusted risks were estimated using multivariable analysis that incorporated age. Risk factors for infection were expressed as odds ratio (OR) and 95% confidence interval (CI). A two-sided *p*-value <0.05 was considered statistically significant.

## Results

**Figure 1:**
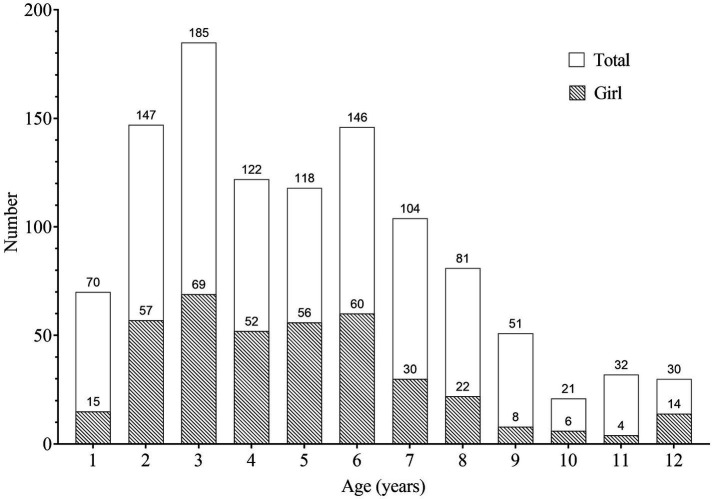
Distribution of patients by age.

The study included 1,107 children, comprising 714 (64.5%) males and 393 (35.5%) females, with a male-to-female ratio of 1.8–1. Median age was 5 years (IQR, 3–7). Infants and toddlers (0–3 years) accounted for 402 (36.3%) cases, pre-school children (4–6 years) 386 (34.9%) cases, and school-age children 319 (28.8%) cases (including 7–9 years 236 cases, 10–12 years 83 cases). The number of children aged over 3 years gradually decreased with increasing age (Figure 1). The proportion of female children in school age decreased with increasing age. In toddlers and pre-school children, the proportion of female patients (39.2%) was significantly higher than that in school-aged children (26.3%), with an OR of 1.81 (95% CI, 1.35–2.41, *p* < 0.001).

### Location of injury

Most children were injured outdoors, totaling 581 (52.5%) cases, with 526 (47.5%) cases occurring indoors. Among the children injured outdoors, 384 (66.1%) were male and 197 (33.9%) were female. The proportion of outdoor injuries increased with age. The outdoor injury proportion was 29.6% among children aged 0–3 years, rising to 80.7% among those over 10 years old.

### Injury sites

**Figure 2:**
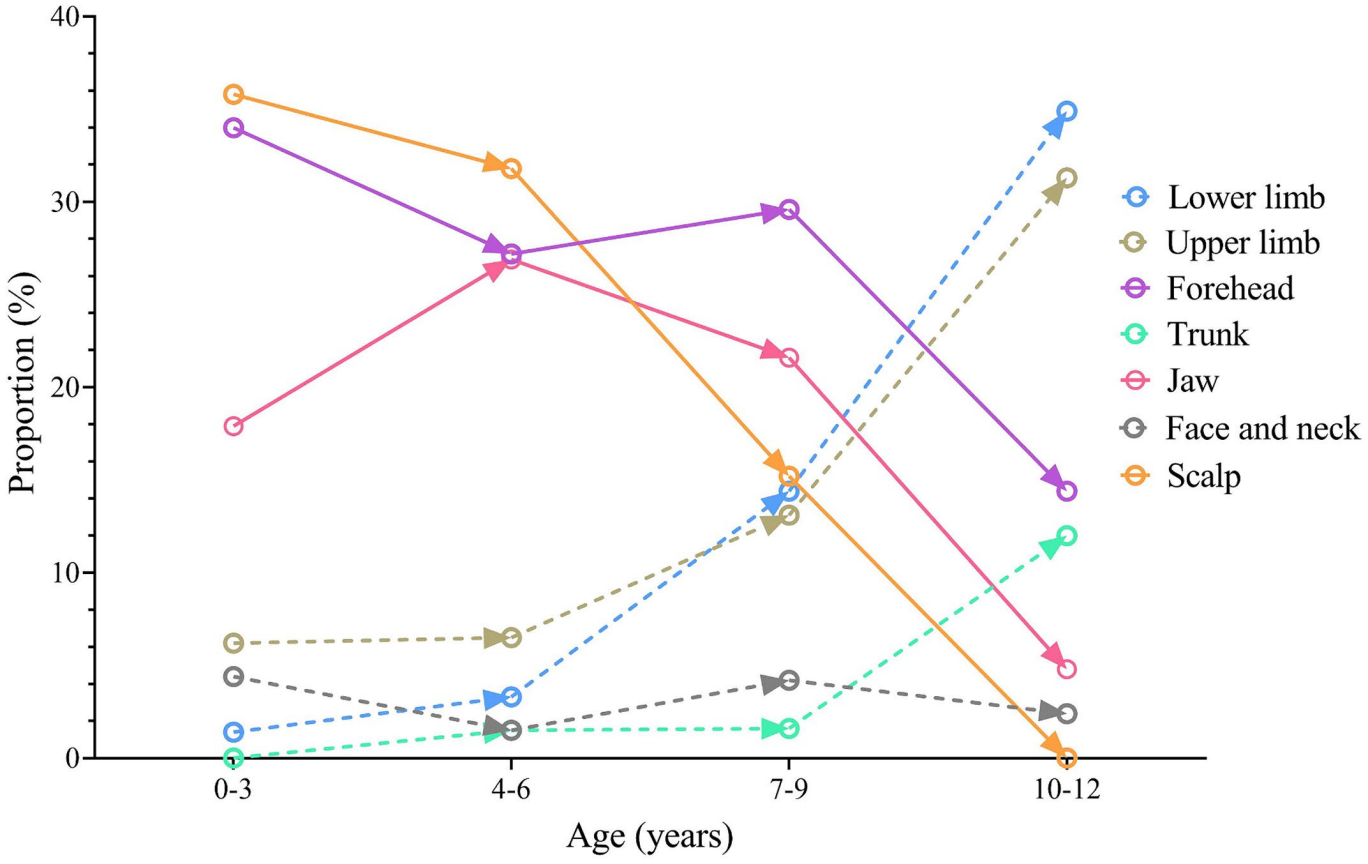
The changing of proportions of different injury sites by age group.

The most common injury site was the forehead, with 324 (29.3%) cases, followed by the scalp with 303 (27.4%) cases, and the lower jaw with 231 (20.9%) cases. The detailed distribution of injury sites is shown in Table 1. The proportion of different injury sites by age group is shown in Figure 2. In infants, toddlers (0–3 years), and pre-school children (4–6 years), scalp, forehead, and lower jaw lacerations were most common. The proportion of these sites significantly decreased among school age children. The proportion of injuries to the upper and lower limbs rose rapidly after 4 years of age, and the risk of injuries to the trunk and perineum increased rapidly after 7 years of age. Facial and neck injuries fluctuated around 3%.

**Table 1:** Characteristics of included children.

Characteristics	Patients (*n* = 1,107)
Age (years), median (IQR)	5 (3.7)
Sex (%)	
Female	393 (35.5)
Male	714 (64.5)
Location (%)	
Outdoor	581 (52.5)
Indoor	526 (47.5)
Body parts (%)	
Scalp	303 (27.4)
Forehead	324 (29.3)
Face and neck	36 (3.3)
Jaw	231 (20.9)
Upper limb	111 (10.0)
Trunk	20 (1.8)
Lower limb	82 (7.4)
Length (cm)	
<1	128 (11.6)
1–3	932 (84.2)
>3	47 (4.2)
Duration (hours)	
<2	466 (42.1)
2–4	576 (52.0)
4–8	54 (4.9)
>8	11(1.0)

### Wound length

The length of most wounds in children was 1–3 cm, accounting for 84.1% (932 cases). Wounds of <1 cm were most common in forehead injuries (37.5%). Most wounds (80.7%) only involved the fat layer, with the deep fascia intact.

### Time from injury to suturing

In this study, the overall time from injury to suturing was short for most children. 42.1% of the children (466 cases) received timely suturing within 2 h of the injury, and 52.0% (576 cases) within 2 to 4 h. 1% of the children (11 cases) had skin lacerations exceeding 8 h at the time of suturing.

### Treatment outcomes

All wound debridements and suturing were performed in the emergency room under local anesthesia. There were no cases of suturing failure. Poor wound healing occurred in 68 cases (6.1%). Among the children with poor wound healing, 54 (79.4%) were injured outdoors, 25 (36.8%) had injuries to the lower limbs, and 14 (20.4%) had wound lengths greater than 3 cm. The rates of poor wound healing were 6.6% for medical professional with less than 2 years of experience, 6.3% for those with 2–5 years, and 5.3% for those with over 5 years.

### Factors affecting poor wound healing

**Figure 3:**
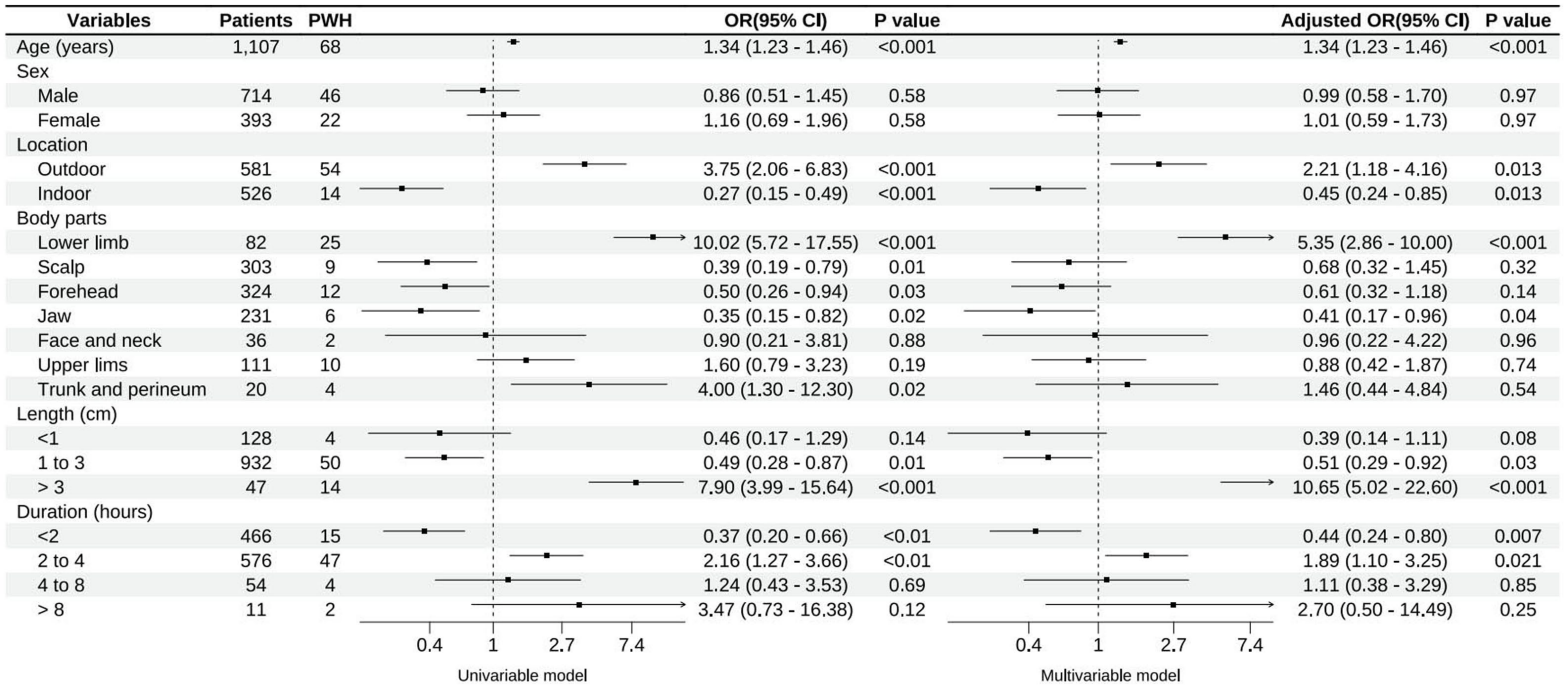
Factors associated with poor wound healing (PWH).

Univariable analysis identified older age, outdoor injuries, lower limb injuries, trunk and perineum injuries, and wound lengths greater than 3 cm as risk factors for poor wound healing. The variations in wound healing rates among the different groups of medical professionals did not reach statistical significance (*p* = 0.80). The risk of poor wound healing increased by 34% for each additional year of age. In the multivariable model, outdoor injuries (OR, 2.21; 95% CI, 1.18–4.16), injuries to the lower limbs (OR, 5.35; 95% CI, 2.86–10.00), and wound lengths greater than 3 cm (OR, 10.65; 95% CI, 5.02–22.60) remained significantly associated with poor wound healing. The results of the univariable and multivariable analyses are presented in Figure 3.

## Discussion

This study, involving 1,107 children, revealed that the occurrence of common pediatric skin lacerations showed clear age distribution characteristics, with age and injury site significantly correlated with poor wound healing. The findings of this study may help in developing targeted injury prevention strategies for children of different ages and optimizing pediatric emergency medical resources. The current study also provides information for predicting the outcomes of common wound treatment.

In this study, the proportion of male children was higher than that of female children, and the proportion of males increased with age. Similar to previous reports on children’s unintentional injuries, this association may be due to boys being more active and prone to taking risks (5, 8). The literature indicates that school-aged children have the highest incidence of accidents, followed by pre-school children and toddlers, with infants having the lowest incidence (8–10). This may vary due to varied definitions of injury and differences in the populations included in different studies. In our study, the majority were infants and toddlers, accounting for 36.3% of all cases, followed by pre-school children with 386 (34.9%) cases. The high incidence of unintentional injuries in infants and toddlers is related to their strong curiosity, restlessness, lack of coordinated movements, and incomplete self-protection awareness (11). During this period, the brain and physical development are not fully mature, leading to a higher risk of falls and injuries, especially to the head. Therefore, toddlers and pre-school children are important demographics for accident prevention, with the head being a primary focus. For school-aged children, preventing unintentional injuries to the upper and lower limbs is important.

In this study, most injuries occurred outdoors. As children grow, their outdoor activity time and intensity increase, possibly leading to more frequent injuries (12, 13). Additionally, outdoor injuries are potential risk factors for poor wound healing. However, after adjusting for age and gender in the multivariable analysis, the effect of outdoor injuries on poor wound healing decreased. This suggests a common effect between age and outdoor injuries, consistent with existing knowledge.

As children’s intelligence and physical development, coordination, and awareness of danger improve, the number of injuries in children aged 4–15 years shows a declining trend (8, 14). The common injury sites also shift from head and face injuries to limb injuries, mostly due to sports and traffic accidents. This is likely due to participation in sports and increased outdoor activity. These findings are useful for parents, schools, and society in formulating targeted prevention strategies for child accidents. For example, regularly holding safety education lectures, developing personalized prevention strategies for different age groups, popularizing first aid knowledge, taking emergency measures after an accident, and creating beneficial conditions for effective and timely treatment (15). Furthermore, to prevent traffic accidents in school-aged children, policymakers, schools, and parents should strengthen road safety awareness, and traffic authorities should enhance safety supervision and management around schools.

Although all children in this study received early treatment, the rate of poor wound healing was higher than previously reported (3–5%) (16). This may be due to different definitions of poor wound healing in various studies. In our study, poor wound healing included wound exudation, wound rupture, scar hyperplasia, sinus formation, and skin or flap necrosis. This category encompasses both superficial and deep wound infections. Although superficial wounds mostly do not require additional surgery, they are often significantly associated with increased scarring, especially on the face, which greatly affects aesthetics (17). In pediatric skin injuries, deep wound infections and ruptures requiring additional surgical intervention are relatively rare.

Many factors can affect wound healing, such as tumors, diabetes, infection, local poor blood supplement, necrotic tissue coverage, etc. (17). Additionally, the proliferation of microbes, the presence of drug-resistant microbes, and the formation of bacterial colonies are important factors hindering wound healing and causing infections. Normally, wound healing in children’s skin is fast, but it is also affected by various factors. Studies have indicated that many factors, including protein-energy malnutrition, infection, inadequate local blood perfusion, edema, and medications, can impede wound healing (18, 19). Our study found that age and injury site were correlated with poor healing. With increasing age, children’s skin blood supply gradually becomes less abundant, leading to slower wound healing and higher risk of infection compared with smaller children. Injuries to the lower limbs are often caused by high-energy trauma, frequently accompanied by contusions to surrounding soft tissues. Additionally, the blood supply to the lower limbs is relatively poorer compared to the head and face, thus leading to a higher rate of poor wound healing.

Other factors associated with wound healing included the types of wound cleaning and dressings employed. Although our study consistently used sterile gauze, literature suggests that the choice of dressing can significantly affect the outcomes of wound healing (20). Different dressing materials, such as hydrocolloids, alginate, and silicone gels, are known to create varying micro-environments around a wound, which can influence both the rate and quality of healing (21). For instance, hydrocolloid dressings maintain a moist environment that can accelerate epithelialization and reduce pain during dressing changes (20, 21). Furthermore, the disinfectants used for wound cleaning, such as iodophor, hydrogen peroxide, and alcohol, plays a critical role in managing the microbial load and preventing infection, which are crucial for optimal healing outcomes (22). Therefore, the selection of appropriate antiseptics and dressing should be tailored to the specific characteristics of the wound to optimize healing.

The goodness-of-fit analysis for our logistic regression model, with an *R*^2^-value of 0.454, indicates that our model, although useful, does not capture the full variability in the outcomes of pediatric skin lacerations. This suggests that additional factors not included in our model could have a significant impact on wound healing. Variables such as the application of antibiotics, the types of disinfectants used, and nutritional status could play crucial roles. These factors were not included in the current study, which could potentially limit the comprehensiveness of our findings. Future studies should consider these variables to provide a more detailed understanding of the factors influencing pediatric skin laceration outcomes.

The current study has several limitations. First, this is a retrospective study, with inherent methodological limitations of retrospective research, such as the potential for selection bias. Second, to enhance clinical applicability and study factors affecting wound healing, this study only included wounds treated by suturing in the emergency department. Most pediatric skin injury could be managed under local anesthesia. Wounds that did not require suturing (e.g., superficial abrasions) and those that could not be sutured (e.g., tissue defects) were not included. Third, being a single-center study, the number of cases included is limited, which might affect statistical power, although many *p* values in the results are lower than 0.05. The generalizability of the conclusions is also limited. Forth, the data was sourced exclusively from a large general hospital that may not mirror the pediatric patient demographics seen in specialized pediatric hospitals. The diversity in disease spectrums and patient demographics between emergency departments in general and specialized pediatric hospital could lead to variations in injury types and treatment outcomes. Fifth, due to the small number of cases, it was not possible to determine whether suturing wounds exceeding 8 h after injury was related to poor healing. In addition, the study did not evaluate the scarring condition of the wounds.

## Conclusion

The composition of common unintentional pediatric skin lacerations shows distinct age-related characteristics. Head injuries are most common in infants and pre-school children, while the proportion of limb injuries rapidly increases in school-aged children. Older age, outdoor injuries, longer wound lengths, and lower limb injuries are risk factors associated with poor wound healing.

## Data availability statement

The raw data supporting the conclusions of this article will be made available by the authors, without undue reservation.

## Ethics statement

The studies involving humans were approved by the Medical Ethical Committee of Qlu Hospital of Shandong University. The studies were conducted in accordance with the local legislation and institutional requirements. The ethics committee/institutional review board waived the requirement of written informed consent for participation from the participants or the participants' legal guardians/next of kin because this was a retrospective study of de-identified patients.

## Author contributions

HG: Conceptualization, Data curation, Formal analysis, Writing – original draft. YL: Methodology, Resources, Validation, Writing – review & editing. SJ: Software, Validation, Visualization, Writing – original draft. WZ: Investigation, Methodology, Resources, Writing – review & editing. YG: Conceptualization, Data curation, Software, Writing – review & editing. LP: Investigation, Project administration, Validation, Visualization, Writing – review & editing.
